# Defining the Emergence of New Immunotherapy Approaches in Breast Cancer: Role of Myeloid-Derived Suppressor Cells

**DOI:** 10.3390/ijms24065208

**Published:** 2023-03-08

**Authors:** María Luisa Sánchez-León, Carlos Jiménez-Cortegana, Silvia Silva Romeiro, Carmen Garnacho, Luis de la Cruz-Merino, Daniel J. García-Domínguez, Lourdes Hontecillas-Prieto, Víctor Sánchez-Margalet

**Affiliations:** 1 Laboratory Service, Department of Medical Biochemistry, Molecular Biology and Immunology, School of Medicine, Virgen Macarena University Hospital, University of Seville, 41009 Seville, Spain; 2 Oncology Service, Virgen Macarena University Hospital, Department of Medicine, School of Medicine, University of Seville, 41009 Seville, Spain; 3 Department of Normal and Pathological Cytology and Histology, School of Medicine, University of Seville, 41009 Seville, Spain

**Keywords:** breast cancer, tumor microenvironment, myeloid-derived suppressor cells, immunotherapy

## Abstract

Breast cancer (BC) continues to be the most diagnosed tumor in women and a very heterogeneous disease both inter- and intratumoral, mainly given by the variety of molecular profiles with different biological and clinical characteristics. Despite the advancements in early detection and therapeutic strategies, the survival rate is low in patients who develop metastatic disease. Therefore, it is mandatory to explore new approaches to achieve better responses. In this regard, immunotherapy arose as a promising alternative to conventional treatments due to its ability to modulate the immune system, which may play a dual role in this disease since the relationship between the immune system and BC cells depends on several factors: the tumor histology and size, as well as the involvement of lymph nodes, immune cells, and molecules that are part of the tumor microenvironment. Particularly, myeloid-derived suppressor cell (MDSC) expansion is one of the major immunosuppressive mechanisms used by breast tumors since it has been associated with worse clinical stage, metastatic burden, and poor efficacy of immunotherapies. This review focuses on the new immunotherapies in BC in the last five years. Additionally, the role of MDSC as a therapeutic target in breast cancer will be described.

## 1. Introduction

Breast cancer (BC) is the leading cause of cancer death in women worldwide and a global public health problem due to its high incidence. It is estimated that 2.1 million cases of BC were diagnosed in the last years, which means that 1 out of 4 women was diagnosed with the disease [1]. The World Health Organization estimated an increase in the incidence of BC of more than 2.5 million new cases between 2018 and 2040 as well as an increase of more than 800,000 deaths in this time frame [2,3]. Breast tumors are highly heterogeneous and can be classified according to molecular and hormonal profiles, expression of growth factors, and other biomarkers [4]. According to the gene expression profile using Prosigna Breast Cancer Prognostic Gene Signature Assay, there are five intrinsic subtypes: luminal A, luminal B, human epidermal growth factor receptor 2 (HER2) enriched, basal-like/triple-negative breast cancer (TNBC), and claudin-low [3,4,5,6]. This clustering is critical for clinical management, treatment decisions, and patient care.

Luminal tumors represent approximately 70% of all invasive BCs [4] and are characterized by estrogen receptor (ER) and/or progesterone receptor (PR) positivity and HER2 negativity. Specifically, luminal A is a low-grade subtype of BC with a low ki67 index, and clinically manifests slow growing, which ultimately leads to better prognosis, whereas luminal B expresses higher grade and Ki67 index and is associated with worse prognosis. A subset of luminal A and luminal B BCs shows HER2 amplification/overexpression [3,5,6,7,8,9,10].

HER2-enriched BCs account for 10–15% of diagnosed tumors and do not express ER or PR. Additionally, this type of BC has a high expression of genes and proteins, such as the *ERBB2/HER2neu* gene and the growth factor receptor bound protein 7, which are related to proliferation, angiogenesis, and invasion of tumor cells, hence HER2-enriched BC patients have lower progression-free survival (PFS) [3]. Basal-like/TNBC has a diagnosis rate of 20%, and does not express ER, PR, or HER2 [6,7,8,9,10], but a high expression of genes related to cell proliferation and a high number of mutations can be observed in the genome, such as the *breast cancer gene 1* mutation, which is found in 80% of TNBCs. In turn, TNBC can be divided into six different subtypes, although the impact of this classification in treatment decision is not completely clear and still needs further research: basal-like 1 and 2, mesenchymal, mesenchymal stem-like, immunomodulatory, and luminal with androgen receptor expression [8,11,12,13].

Similarly, claudin-low BCs have poor prognosis and are negative for ER, PR, and HER2. They account for 7–14% of all invasive tumors and are characterized by a low expression of critical molecules in the cell–cell adhesion (including claudins 3, 4, and 7, occludin and E-cadherin) and have a high infiltration of immune and stromal cells [8].

Given the vast heterogeneity of BCs, treatment choice is rather complex, so different strategies have been developed throughout these years to cure the disease and bring patients the best quality of life [14,15]. In the early setting, luminal A tumors are usually treated with surgery followed by radiotherapy (RT) and endocrine therapy (ET). Chemotherapy (CT) is used for large tumors or nodal involvement. Luminal B BCs are treated with (neo)adjuvant CT followed by surgery, RT, and ET. HER2+ BCs are treated with (neo)adjuvant CT combined with anti-HER2 drugs, followed by surgery and RT. Early stage TNBC is treated with (neo)adjuvant CT followed by surgery and RT [14].

Currently, in the advanced setting, innovative treatments are now available for the different subtypes of BC, including (but not limited to) ET combined with cyclin-dependent kinases 4/6 inhibitors in luminal tumors [16], new anti-HER2 drugs for HER2+ tumors, and immune checkpoint inhibitors combined with CT. Altogether, it may provide the maximum benefit to the patients, minimizing recurrence, treatment resistance, and toxic effects, although combinatorial approaches should be carefully chosen to ensure the best quality of life [17,18]. However, there are 15–20% of patients who recur and die, so effective new therapeutic strategies are needed. In this sense, accumulating data based on clinical and preclinical studies support that immunotherapy improves both the response and outcomes of BC patients compared to standard therapies. Unfortunately, only a small proportion of BC patients benefit from immunotherapy and the treatment response is not uniform due to the heterogeneity of this tumor. Thus, the current efforts try to understand the different responses observed across BC subtypes and identify biomarkers that predict the patient’s outcome. However, it is a challenge that still needs to be met, because immunotherapy responses depend on both tumor immunogenicity and the ability of the immune system to response effectively and eradicate the tumor [17,18]. For all the previous reasons, the aim of this review is to provide an overview of the new immunotherapies in BC as well as the role that the immune system plays, mainly focused on the emerging myeloid-derived suppressor cells (MDSCs), a population of pathologically activated and immature myeloid cells, as a promising therapeutic target in this disease.

## 2. The Role of the Immune System in Breast Cancer

**Figure 1:**
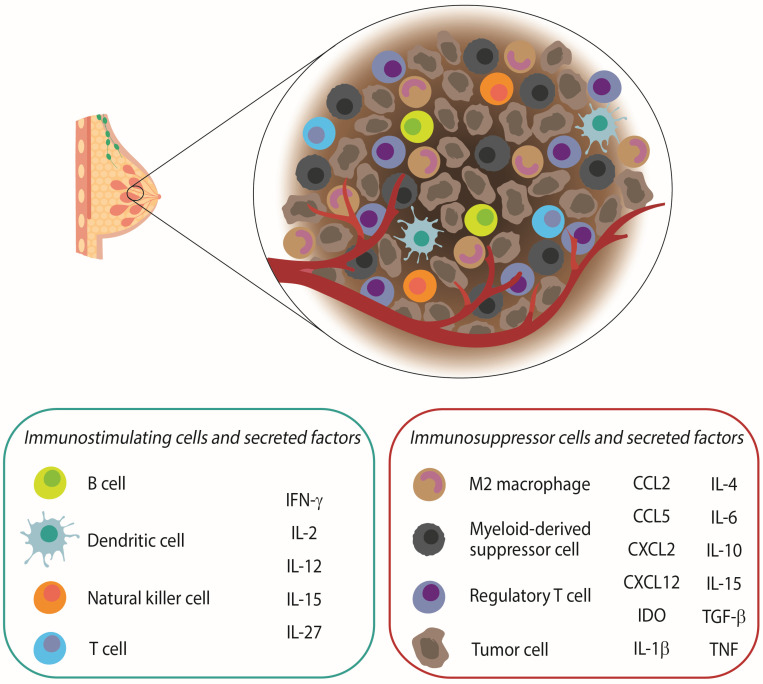
Composition of the tumor microenvironment (TME) in breast cancer. The breast cancer microenvironment is composed by a variety of immune cells, including a huge proportion of suppressive cells, such as M2 macrophages, regulatory T cells, and myeloid-derived suppressor cells that ultimately induce the secretion of proinflammatory mediators to promote the development and progression of cancer cells. However, the TME also includes a low proportion of dendritic cells and tumor-infiltrating lymphocytes, such as T, B, and Natural Killer cells. In this sense, immunotherapies have shown to boost the maturation and function of those cells to exert potent immune responses against the tumor.

The immune system plays a dual role in the biology of BC since it not only promotes tumor growth through different mechanisms that generate chronic inflammation [19,20,21], which is considered a new hallmark of cancer, but also mediates tumor eradication. The relationship between the immune system and BC depends on several factors, such as tumor histology and size, lymph node involvement, as well as the presence of a wide variety of cells, molecules, and chemicals that shape the tumor microenvironment (TME) [22]. The balance between inhibitory and stimulatory signals is decisive to explain the final effect of anergy or activation of the immune system against cancer cells [23]. Nevertheless, mammary tumors have been classically considered to have low immunogenicity due to the heterogeneous expression of antigens in the primary tumor or metastases, modifications in the antigenic profile during progression, low levels of major histocompatibility complex (MHC) expression, release of suppressor cytokines, and expression of T-lymphocyte activation suppressor molecules, such as cytotoxic T lymphocyte-associated antigen 4 (CTLA-4) and programmed cell death protein 1 (PD-1) [23]. All this favors a suppressive microenvironment that helps tumor cells to escape from immune surveillance [24]. Therefore, the TME is a complex network composed of tumor cells; immune cells involved in the innate response, such as natural killer (NK) cells, monocytes, macrophages, dendritic cells (DCs), and MDSCs; cells involved in the adaptive response, such as T helper lymphocytes (Th) 1, Th2, regulatory T cells (Tregs), cytotoxic T cells, B lymphocytes, stromal, endothelial, and epithelial cells; and cytokines [25,26,27]. All this is communicated through molecular pathways, giving rise to a chronic inflammatory response favoring both tumor initiation and progression. This results in an immunosuppressive microenvironment with low antigen expression, loss of co-stimulatory molecules, and MHC class I system, which ultimately promote an increase of immunosuppressive molecules, such as indoleamine 2,3-dioxygenase (IDO), PD-1, CD39, CD73, or adenosine receptors. Interestingly, IDO expression has recently been shown to favor the immunosuppressive status of BC patients with positive nodes. This cytokine, together with transforming growth factor (TGF)-β or interleukin (IL)-10, can be produced by suppressive cell populations, such as MDSCs [28,29,30] or M2-like tumor associated macrophages (TAMs) to promote disease progression in BC patients via bone metastases and epithelial–mesenchymal transition [31,32,33], as shown in Figure 1.

Altogether, it seems clear that the TME plays a major role in tumor initiation, development, and progression of BC cells, which has encouraged the design and development of new immunotherapeutic strategies to treat BC patients in order to avoid the TME resistance against BC treatments [34].

## 3. Immunotherapy in Breast Cancer

BC has traditionally been considered as a cold tumor in terms of immunogenicity. However, much evidence has described immunogenic activity in the BC subtypes [35,36]. This finding is allowing a variety of studies to explore the great potential of different immunotherapies in BC patients [37] to provide predictive and prognostic information, and to optimize standard therapies (surgery, RT, CT, and ET), which has resulted in improved clinical outcomes with a significant improvement in survival rates [38]. However, increasing BC immunogenicity and modulating the TME are some necessary strategies for improving therapeutic efficacy.

Immunotherapies can be considered as passive or active. Passive immunotherapies include anti-HER2 targeted monoclonal antibodies, such as trastuzumab, pertuzumab, and margetuximab; whereas active immunotherapies mainly encompass cancer vaccines to boost the antitumor response by activating autologous immune cells and inducing a therapeutic effect [37].

### 3.1. Vaccines

The immunotherapeutic effect of vaccines is to activate both the adaptive immune response and immunological memory, destroying tumor cells with minimal toxicity [37]. Vaccines are formulated with different types of antigens, which are recognized by the immune system to induce beneficial therapeutic effects [39,40,41]. The main antigens in BC are HER2 or HER2-related peptides [42,43], but other non-HER2 antigens are also being studied, including mucins, telomerase reverse transcriptase (hTERT), and p53 [39]. In addition, adjuvants play a vital role since they enhance the antigen immunogenicity and regulate immune responses [44,45,46,47].

There are different types of vaccines depending on the type of antigen. The most studied are those formulated with tumor-associated antigen (TAA) peptides [48], but others such as tumor protein- or carbohydrate-associated antigens, and antigens based on DNA and DCs are also being studied to stimulate both innate and adaptive antitumor immunities [39,49,50,51].

#### 3.1.1. Peptide Vaccines

E-75 vaccines are based on HER2 as the main TAA in BC. Different HER2-derived immunogenic peptides have been studied, with different specific immune responses [37]. Neuvax is the most studied BC vaccine and is formulated with the E75 peptide, derived from HER2, recognized by MHC class I, and combined with the immune adjuvant granulocyte-macrophage colony-stimulating factor (GM-CSF). The great advantage is that it stimulates CD8+ cytotoxic and memory T cells, which bind human leukocyte antigen (HLA) molecules on APCs to recognize, neutralize, and destroy HER2-expressing primary tumor cells and metastatic tumors [52,53]. Preclinical studies were initially conducted to analyze the immunogenicity of the E-75 peptide [54,55,56,57], followed by phase I trials such as NCT00841399, in which patients with metastatic BC and different HER2 expression were enrolled, and both doses and inoculation routes were optimized. This vaccine proved to be safe and effective in stimulating E-75 specific CTLs [58].

This peptide is HLA-restricted, hence HLA-A2 or HLA-A3 positive patients were vaccinated, while those who were negative were followed up as unvaccinated controls. Increasing doses of the vaccine were administered monthly, for 4–6 months, with good tolerance and minimal toxicity for different doses. Phase II trials (such as NCT02297698) demonstrated improved survival rates after vaccination, with acceptable tolerance and limited toxicity. Neuvax finally completed a phase III trial, which evaluated the vaccine in combination with GM-CSF vs. placebo plus GM-CSF to prevent recurrence in BC patients with node positive, HER2 low expression, and HLA-A2+/A3+ (NTC01479244). For women with ductal carcinoma in situ, a phase II clinical trial is being conducted to evaluate the efficacy of Nelipepimut-S plus GM-CSF or Sargramostim (Leukine^®^) (NCT02636582) [59,60].

Another immunogenic peptide used in BC vaccines is GP2, a fragment of the HER2 transmembrane domain with nine amino acids in length. GP2 binds HLA-2 with less affinity than E-75 and also activates CTLs [61]. A phase I trial (NCT03014276) conducted in lymph node-negative patients resulted in the formulation of GP2 plus GM-CSF, being well tolerated and safe. The phase II trial NCT00524277 was conducted in high-risk patients with positive lymph nodes, HER2 overexpression, and free of disease, demonstrating promising safety and good tolerance [43,54]. Currently, a phase III trial is ongoing to demonstrate the efficacy of this treatment. Finally, AE37 is a peptide that consists of 15 amino acids and activates Th lymphocytes [62]. In the phase II trial NCT00524277, patients with HER2 expression at all stages of disease were enrolled, showing no statistical significance on disease-free survival after using this type of vaccination [39,63].

In other types of BC vaccines, telomerase is involved. This ribonucleoprotein has polymerase activity to maintain telomere length and leads to cellular immortalization, so its aberrant expression is associated with increased survival and proliferation of tumor cells [38,64]. It is overexpressed in most tumors, including BC [38,39,65]. The synthetic hTERT DNA vaccine, INO-1400, is composed of a plasmid encoding for the catalytic subunit of hTERT with two immunogenic differentiating mutations that elicit a broad CTL-mediated immune response against tumor cells. Yan and colleagues demonstrated the potent immunity exerted by this vaccine in preclinical murine and primate models [37]. In mice, hTERT generated a strong immune response characterized by the increase of CD107a-, interferon (IFN)ɣ-, and tumor necrosis factor (TNF)α-producing cells [38,66]. In monkeys, slower tumor growth and longer overall survival (OS) were achieved, and minimal side effects upon administration were observed [66]. In the phase I clinical trial NCT02960954, INO-1400 was administered by intradermal injection alone or with an immune system activator, IL-12 (INO-9012), in patients with breast, lung, and pancreatic cancers at high risk of relapse after surgery and adjuvant therapy. In this study, dose escalation was carried out to assess the safety and tolerability of the vaccine [37]. Other clinical trials are underway, in both metastatic (NCT00573494 and NCT01660529) and adjuvant (NCT02960594 and NCT00753415) settings [39]. 

BC vaccine constitutes a new therapeutic strategy to enhance anti-cancer immunity. Current results show that vaccines are safe, although this immunotherapy still needs clinical verification. In addition, there are several limitations. One of them is the need for a suitable adjuvant to generate an effective immune response. It is restricted to a few epitopes, resulting in a limited response against tumor cells. Furthermore, peptides have a short half-life, poor enzymatic stability, and high clearance rates. Some studies are trying to solve these limitations using multivalent synthetic long peptides, containing epitopes that can be recognized by MHC class I and II in order to enhance the immune response of these vaccines by activating both CD8+ and CD4+ T lymphocytes [63].

#### 3.1.2. Protein-Based Vaccines

These vaccines integrate a protein fragment of the tumor antigen HER2, whose amino acid sequence is longer than that of the peptides. Protein-based vaccines utilize both the intra- and extracellular domains of the HER2 receptor. Interestingly, these vaccines can uptake, process, and present multiple epitopes to MHC I and II, thus they are not HLA-restricted vaccines [43]. Additionally, they significantly activate T cells, resulting in an increased immune response [67,68]. The first clinical study carried out was a phase I trial in which the vaccine integrated the intracellular domain of HER2 plus GM-CSF [69] to evaluate the immunogenicity in patients with HER2-overexpressing breast and ovarian cancers who were in complete remission after being treated, resulting in a promising tolerance [63], immunity maintenance [43], and HER2/neu-specific immunoglobulin G antibody immunity. Another study conducted by Hamilton et al. aimed to evaluate the immunogenicity, safety, and effect of the anti-HER 2 protein [27] using a vaccine consisting of a recombinant protein combined with Lapatinib in patients with HER2-overexpressing metastatic BC and refractory to trastuzumab, which demonstrated an improved survival in BC patients (92%) after 300 days [70]. Another phase I clinical trial (NCT00058526) used a recombinant HER2 protein and AS15 as an adjuvant and was administered to 61 previously untreated patients with trastuzumab in stage II-III after surgical resection in the adjuvant setting [71]. An association was found between the dose administered, vaccination schedule, and prevalence of HER2-specific humoral responses. This immunity was maintained for more than 5 years in six out of eight patients who received the highest dose. In 40 metastatic patients, the same vaccination regimen was administered in the first or second line of treatment, after trastuzumab maintenance, which was well tolerated and showed promising clinical activity, with two objective responses and 10 patients with stable disease [72]. These vaccines are still in the early stages of research and further clinical trials are needed to increase the number of patients recruited, which would probably provide more data on the impact on survival, immunization efficacy, and long-term follow-up [73].

#### 3.1.3. Tumor Cell Vaccines

Whole tumor cells or cell lysates are also used to stimulate immune responses. Antigens are derived from the patient’s own autologous tumor cells. However, this is a very complex, expensive method and allogeneic cells are used as an alternative [72]. They can be manipulated to express cytokines or chemokines to maximize the immune response. As an adjuvant, GM-CSF stimulates the migration of DCs, T cells, eosinophils, and macrophages to the injection site [59,74], which trigger a polyvalent immune response. However, tumor cell vaccines contain endogenous antigens, may cause autoimmune reactions, and a standardized method for the preparation of such vaccines is still lacking [43]. Different studies have explored the efficacy of these vaccines in BC patients. One of them involved 121 patients diagnosed with breast and ovarian cancers, who received a vaccine formulated with autologous tumor cells transfected with Newcastle disease virus [59,75]. The OS at 4 years was 96%, which validated the efficacy of the vaccine [75]. In another study, 42 patients received a vaccine composed of autologous and allogeneic cells, three TAAs combined with GM-CSF and IL-2, which resulted in a significant increase of lymphocyte proliferation in more than 50% of patients [59,76]. These vaccines might be administered safely to patients, improving the immunity and clinical efficacy. Currently, two completed clinical trials have demonstrated the effectiveness and safety of these vaccines in BC patients (NCT00317603 and NCT00880464). However, the manufacturing process is expensive and there may be a high tumor antigenic variability [59].

#### 3.1.4. Dendritic Cell-Based Vaccines

DCs are highly specialized cells whose main function is to modulate the primary immune response via antigen presentation to CD4+ and CD8+ T lymphocytes [77,78], so there is an interest in using this cell population to generate vaccines. Blood from cancer patients contains immature DCs supplied with TAA encoding recombinant DNA/RNA, and DC-tumor hybrids that are stimulated with specific cytokines to induce maturation. Those cells are currently in use to generate vaccines. Kugler et al. demonstrated the efficacy of these vaccines in breast and ovarian cancer patients [79]. Avigan et al. fused tumor cells from autologous DC patients to generate the cell-based vaccine that ultimately generated strong immune responses in breast and renal cancer patient [80]. Zhang et al. generated a BC vaccine by fusing DCs with TNBC cells, resulting in a potent anti-tumor immune response by facilitating lymphocyte proliferation [81]. Preclinical studies for the development of DC vaccines for BC have also shown promising results: Sakai et al. modified DCs by transducing them with a non-signaling neu oncogene which resulted in no mammary tumor development in BALB-neu transgenic mice [82].

#### 3.1.5. DNA-Based Vaccines

These types of vaccines use DNA sequence encoding tumor antigens that are usually delivered as plasmids or vectors [43], most commonly from bacterial, cytomegalovirus (CMV), or chimeric promoter SV40 (simian virus 40) -CMV origin [83,84]. The most important aspects are the selection or design of a potent plasmid, an efficient delivery system, and monitoring the immune response after vaccination. The most targeting antigens used are oncoproteins HER2/neu and mammaglobin A (Mam-A). In this regard, there are two trials that studied the effect of such vaccines, observing significant humoral responses. Norell et al. conducted a pilot clinical trial with eight patients diagnosed with metastatic BC. The vaccine was formulated with HER2/neu, low-dose IL-2, and GM-CSF. They observed a strong humoral response, but T-cell responses remained unchanged [85]. Kim et al. set up a phase I clinical trial in which a DNA vaccine composed of Mam-A cDNA was administered to 15 Mam-A positive cancer patients and the immune response was monitored for 6 months. Seven out of 15 patients showed an increase in inducible costimulator (ICOS^Hi^) CD4+ T cells and a decrease in Foxp3C CD4C T cells. Activated ICOS^Hi^ CD4+ T cells expressed INFɣ instead of IL-10, which resulted in lysis of Mam-A expressing tumor cells [86]. These studies showed that DNA-based vaccines are efficient to control the disease, although the safety and immunogenic mechanisms of such vaccines need further research [63,72].

#### 3.1.6. Carbohydrate Antigen Vaccines

One of the disaccharide carbohydrates is Sialy-Tn (STn), which is expressed in a wide variety of tumor cells associated with MUC-1. Due to the good results observed in animal models regarding tumor regression and survival, a synthetic conjugate vaccine was developed, commercially named Theratope^®^ (STn-keyhole limpet hemocyanin—KLH). In a double-blind, phase II clinical trial, 1028 patients with metastatic BC from 126 centers of 10 countries were randomized to receive STn-KLH or KLH alone, co-administered with low-dose cyclophosphamide to increase immunogenicity [87]. The primary endpoint was time to progression (TTP) and OS. Despite the significant levels of STn-specific antibodies observed, there was no improvement in TTP or OS [88]. However, there might be a lack of eligibility criteria, since only 30–40% of breast tumors expressed STn and this was not considered when including patients. In a subgroup analysis, the vaccine arm had better TTP and OS outcomes when ET was administered, indicating that the combination of the STn-KLH vaccine with ET could improve clinical outcomes [88,89]. In summary, vaccines are currently a therapeutic strategy under investigation that have shown promising results, although a better understanding of the TME, immunosuppressive, and tumor evasion pathways is still needed [90]. There is a necessity to conduct clinical trials with larger cohorts of patients, and explore the combination with other treatments, such as checkpoint inhibitors or anti-angiogenic drugs [63]. In addition, vaccines seem to be more useful in patients with minimal disease and, on the contrary, they appear to be ineffective in the metastatic setting [91].

### 3.2. Monoclonal Antibodies (mAbs)

Immunotherapy with mAbs has improved the therapeutic arsenal in the fight against BC, specifically in HER2 receptor overexpressing tumors [92,93]. Trastuzumab (Herceptin^®^) was the first humanized mAb approved by the FDA in 1998 for the treatment of HER2+ metastatic BC in combination with CT. Trastuzumab directly targets the extracellular domain of the HER2 receptor, and several mechanisms of action have been described: the first one involves HER2 receptor degradation by binding its extracellular transmembrane domain, inducing HER2 internalization and degradation through a E3 ubiquitin ligase (c-CBL) [94,95]; the second mechanism of action is to attract cytotoxic innate immune cells to the TME by binding the fragment crystallizable region (Fc region) of IgG1 (Trastuzumab) to the FcɣRIII/CD16 of NK cells, so-called “antibody-dependent cellular cytotoxicity” (ADCC) [96,97].

NK cells release proinflammatory cytokines, such as INFɣ and TNFα, during ADCC, a mechanism known as antibody-dependent cytokine release (ADCR) [97]. The crosstalk among NK cells, tumor cells, and the pro-inflammatory environment induced by ADCC may promote other immune cell populations, which has been called the “vaccination effect” [98]. Finally, the extracellular binding of trastuzumab to HER2 leads to the inhibition of the RAS/MAPK and PI3K/AKT signaling pathways [94,99]. Several studies have described its role in immune system activation, including an increase of TILs, which has been associated with a decrease in distant recurrence [100]. Others reported that TILs had prognostic and predictive value as it improves the pathological complete response (pCR) and disease-free survival (DFS) [101,102]. Bense et al. showed that the presence of different types of immune cells differed according to BC subtype [103]. A high Treg fraction in HER2+ tumors was associated with a lower rate of pCR, DFS, and OS [104]. Increased Tɣδ lymphocytes in all BC patients was associated with a higher rate of pCR, prolonged DFS, and OS. High levels of activated mast cells were associated with worse prognosis, DFS, and OS in HER2+ patients [97]. Although it is a standard drug in targeted therapy against HER2, it should be noted that between 27% and 42% of patients develop de novo and acquired resistance to it in neoadjuvant and adjuvant therapy, which hinders its clinical benefit. This resistance mechanism involves the TNFα signaling pathway, which is also induced by the expression of mucin 4, a protein that masks the trastuzumab binding epitope on the HER2 receptor to promote the spread of tumor cells, and which is a biomarker of poor response to adjuvant trastuzumab [99]. HLA-G expression in tumor cells has been identified as another mediator of trastuzumab resistance, which, when coupled to the HLA-G/KIR2DL4 interaction, enhances the vulnerability of HER2+ breast tumors to trastuzumab treatment in vivo [105].

Pertuzumab (Perjeta^®^) is a dual HER2/HER3 mAb that was approved by the FDA in 2012 in combination with trastuzumab and docetaxel for first-line treatment of HER2+ metastatic BC as it increased OS, according to the results obtained in the Cleopatra trial (NCT00567190) [95,105]. It was subsequently approved in the early disease setting, based on the results from the NeoSphere (NCT00545688) and Aphinity (NCT01358877) trials. Pertuzumab binds to a different epitope within the extracellular domain of HER2 than trastuzumab, preventing ligand-dependent HER2/HER3 heterodimerization by inhibiting the PI3K and MAPK pathways [95,106,107]. An in vitro study showed that both trastuzumab and pertuzumab alone activated ADCC with equal potency. However, no increase in ADCC activity was observed when administered combined. In vivo studies have shown that the combinatory treatment increased NK migration into the TME, which would delay trastuzumab resistance in BC xenograft models [95].

Margetuximab (MGAH22) is a chimeric mAb with anti-HER2 activity [108] whose fragment antigen-binding (Fab) portion shares the same HER2 specificity as trastuzumab, while the Fc portion is engineered by glycosylation, which increases and improves affinity for the FcɣRIIIa receptor, contributing to improved antibody-dependent cellular cytotoxicity [96,109]. The Fc-independent properties of margetuximab are similar to trastuzumab, including the same binding affinity for HER2+ expressing tumor cells, hence having similar antiproliferative activity. It has been shown that margetuximab is more effective as an ADCC mediator than trastuzumab, both in vitro and ex vivo. In vitro studies suggest that margetuximab promotes greater NK cell activation, expansion, and proliferation than trastuzumab and pertuzumab [96]. In terms of adaptive immune responses, increases in B-cell-mediated HER2-specific antibody levels were found in 42–69% of trastuzumab-treated patients and 94% of margetuximab-treated patients. In addition, increases in T-lymphocyte-mediated responses were observed in 50–78% of trastuzumab-treated patients and 98% of margetuximab-treated patients [96]. In a phase I trial (NCT01148849), 66 patients with HER2-overexpressing advanced BC received intravenous infusion of MGAH22, which was well tolerated, and partial responses and stable disease were observed in 12% and 50% of patients, respectively; also tumor reduction was observed in over half (18/23, 78%) of response-evaluable patients with BC [110].

Finally, margetuximab was approved by the FDA in 2020 in patients with HER2+ metastatic BC who received two or more regimens of anti-HER2 therapy. This approval was based on the SOPHIA study (NCT02492711), a phase III trial that compared margetuximab plus CT versus trastuzumab plus CT in 536 patients with HER2+ metastatic BC who had received at least two prior anti-HER2+ therapies [108,111]. Efficacy analysis using FcɣRIIIa-158 allele expression in 506 patients showed beneficial results with margetuximab on PFS versus trastuzumab in FcɣRIIIa-158F carriers. In contrast, no benefit with margetuximab was observed over trastuzumab in FcɣRIIIa-158V homozygotes [96,111].

Zanidatamab (ZW25) is a biospecific antibody that binds two different epitopes on HER2, HER2 extracellular domains (EDC2 and ECD4) [108]. As a result of these modifications, ZW25 shows a more specific binding to tumor cells, thus inhibiting both ligand- and ligand-independent tumor growth and enhancing receptor internalization and degradation compared to trastuzumab [108]. In vitro assays demonstrated that ZW25 leads to a concentration-dependent ADCC, causing lysis in 52% of HER2-expressing TNBC cell lines (0/1+). In several HER2-expressing cell lines, both activity and synergy with different chemotherapeutic agents such as taxanes, platinums, microtubule inhibitors, and DNA synthesis inhibitors have been observed [98]. Forty-two patients were enrolled in a phase I basket trial (NCT02892123) and 71% received an average of five lines of anti-HER2 treatment for metastatic disease and 20 patients had locally advanced or unresectable. Single-agent anti-tumor activity and a good safety profile were observed after using this molecule. Overall, partial response rate was obtained in 33% of patients and disease control rate in 50%. The most common toxicity was grade 1–2 diarrhea and infusion reaction [98,108]. A clinical trial of ZW25 with palbociclib plus fulvestrant (NCT04224272) is currently ongoing. 

Research in the field of modified mAbs is booming, as they are considered as a promising treatment in the fight against cancer. This is because mAbs can directly target tumor cells to kill them (target therapy) while promoting a long-lasting immune system response. Thus, a considerable number of them, P95HER, MM111, HER2/CD3, among others, are still in preclinical and clinical research with promising results [98].

### 3.3. Antibody Drug Conjugates (ADC)

ADCs are a complex class of drugs designed to deliver antineoplastic drugs in a most accurate and selective way [112]. They consist of a recombinant humanized antibody that covalently binds to a cytostatic agent via a linker that couples the cytostatic to the antibody. This structure combines the potency of small cytostatic molecules with the high specificity of mAbs, targeting specific tumor antigens [113]. Currently, ADCs approved for the treatment of BC target the HER2 receptor and the human trophoblast cell-surface antigen 2 (TROP-2), which is overexpressed in several epithelial neoplasms, especially in TNBC [95]. HER2-targeted ADCs induce tumor cell apoptosis through two mechanisms: ADCC [98] and complement-dependent cytotoxicity (CDC) [114]. Two ADCs targeting the HER2 receptor, trastuzumab emtansine (T-DM1), and trastuzumab deruxtecan (T-Dxd), are currently approved by the FDA.

Trastuzumab emtansine is formed by humanized trastuzumab mAbs conjugated by a non-cleavable thioether linkage to DM1, a derivative of the natural maytansinoid toxin, which inhibits tubulin polymerization leading to cell death. The drug-to-antibody ratio (DAR) is 3.5 [112,115], matching with the average number of drugs conjugated with the antibodies, which is an important attribute of ADCs. T-DM1 was the first ADC approved by the FDA in 2013, supported by the results of the EMILIA trial (NCT00829166), which demonstrated increased PFS and OS in patients treated with this ADC compared to patients treated with lapatinib plus capecitabine for metastatic disease. Similarly, T-DM1 was later approved by the FDA in the adjuvant setting due to the results of the KATHERINE study (NCT01772472), in which T-DM1 patients with residual disease after neoadjuvant trastuzumab therapy were treated. This drug is still being studied in other disease settings and combined with other anti-HER2 drugs, such as tucatinib, a recently approved tyrosine kinase inhibitor. Two clinical trials are still ongoing, CompassHER2-RD (NCT03975647) [112,115] and HER2CLIMB (NCT03975647) [116,117].

T-Dxd is a humanized anti-HER2 IgG1 mAbs (MAAL-9001) with the same amino acid sequence as trastuzumab, bound by a cathepsin cleavable linker, a maleimide tetrapeptide, to the cytostatic exatecan derivative MAAA-1181a (Dxd), a DNA topoisomerase I inhibitor. Upon binding to HER2, T-Dxd internalizes, releases the cytostatic, and causes DNA damage and cell death by apoptosis [118]. It has a potentially greater anti-tumor effect than T-DM1 due to a DAR of 8. This drug was tested in both in vivo and in vitro models, where the pharmacological activities of T-Dxd were evaluated in comparison to those of T-DM1, in HER2-positive cell lines and patient-derived xenograft (PDX) models. This confirmed the higher membrane permeability to Dxd compared to DM1 and also greater stability in plasma, as well as potent antitumor effects in cancers with low HER2 expression, which did not occur for T-DM1 [112]. In 2019, the FDA granted the approval of T-Dxd for patients with HER2+ metastatic BC who had received two prior lines of anti-HER2-based therapy in the metastatic setting, based on the results of the DESTINY-Breast 03 trial (NCT0352910). There are currently several phase Ib/II clinical trials to test this molecule for HER2+ and HER2 low disease, both early stage and metastatic. In the case of HER2 low, the current ongoing trials are the DESTINY-Breast 04 (NCT03734029) and the DESTINY Breast 06 (NCT04494425) studies [112,113,114,115,116,117,118,119]. 

Trastuzumab docarmazine (SYD985) and disitamab vedotin have demonstrated efficacy against tumor cells and are also being tested for efficacy and safety in clinical trials in HER2+ metastatic disease (NCT03262935, NCT03500380). SYD985 has been found to be effective in tumors with both high and low HER2 expression [112,113,114,119].

Sacituzumab-govitecan (Trodelvy) is a treatment to target TROP2, expressed in some tumors including BC. It is involved in promoting cell proliferation, survival, and invasion. In vitro data indicate that cell lines overexpressing this protein are very sensitive to Trodelvy [120,121]. This is the first anti-TROP-2 ADC in its class, composed of humanized mAbs, a cleavable linker that binds the cytostatic SN-38, an active metabolite of Irinotecan, a topoisomerase I inhibitor [52]. It was recently approved by the FDA for the treatment of metastatic TNBC that had received at least two prior therapies for metastatic disease, based on the results of the ASCENT clinical trial, IMMU-132-01 (NCT01631552) [116,121,122]. This molecule improved both survival and objective response rates (ORRs) compared to CT in patients who were not initially diagnosed with TNBC [123]. There are a large number of clinical trials with this molecule both in early disease in the neoadjuvant and adjuvant setting (NCT04230109) and in combination with other drugs such as immune checkpoint inhibitors (NCT04448886; NCT04468061; NCT03424005) and Poly (ADP-ribose) polymerase inhibitors (NCT040392230; NCT03992131) [112,121].

Ladiratuzumab-vedotin (SGN-LIV1A) is a humanized mAb targeting zinc transporter (LIV-1), expressed in TNBC and luminal BC, prostate cancer, and melanoma [112]. This antibody is bound by a cleavable linker conjugated to a potent cytostatic microtubule disrupting agent, thereby inducing tumor cell apoptosis [124]. There are several ongoing phase-I clinical trials, such as NCT01969643, examining safety, tolerability, pharmacokinetics, and anti-tumor activity in patients with LIV-1 positive metastatic BC. Other studies combine SGN-LIV1A with pembrolizumab (NCT03310957) and atezolizumab (NCT03424005) [112].

Glembatumumab-vedotin (GV) is an ADC consisting of a human glycoprotein non-metastatic B (gpNMB) specific IgG2 antibody linked by a linker to a monimethyl auristatine E (MMAE). The gpNMB protein is overexpressed in approximately 40% of TNBCs and is associated with poor prognosis [125]. Preclinical studies have implicated this protein in invasion, metastasis, and angiogenesis [126,127]. Two clinical trials were conducted with this molecule with the main purpose to know its effectiveness: the EMERGE study (NCT01156753) and the METRIC study (NCT01997333), which enrolled patients with advanced TNBC expressing the gpNMB protein [128].

Patritumab deruxtecan consists of a humanized IgG1 anti-HER3 mAb covalently linked to a cytostatic topoisomerase I inhibitor, derived from exatechin, by a tetrapeptide-based cleavable linker. The results of the JapicCTI-163401 clinical trial (NCT02980341) demonstrated promising antitumor activity in previously treated patients with metastatic BC and HER3 expression [112].

The lack of effective therapy for TNBC has encouraged research into new treatments with different therapeutic targets. The “recepteur d’origine nantais” (RON), which belongs to the tyrosine kinase receptor of the MET proto-oncogene family, is involved in the pathogenesis of TNBC and its expression has prognostic value [128,129,130]. Two anti-RON mAbs, Zt/g4 and PCM5B14, have been selected for the development of anti-RON ADCs as cytostatic compounds with different mechanisms of action. Maytansinoid derivate 1 (DM1), MMAE and duocarmycin (DCM) have been conjugated to generate ADCs, such as Zt/g4-MMAE and PCM5314-DCM [128,129,130]. Preclinical studies have shown the therapeutic superiority of anti-RON ADCs, suggesting those drugs as novel therapies for the future treatment of TNBC [129,130,131].

Of note, another molecule, the leucine-rich repeat containing protein (LRRC15), has become a promising anticancer agent due to its overexpression in both the stroma and tumor-associated fibroblasts (CAFs) of some tumors, such as sarcoma, glioblastoma, melanoma, BC, among others [132,133]. Tumor-specific ADCs with LRRC15 expression, such as ABBV-085, are currently under testing in phase I clinical trials (NCT02565758) in sarcomas and other solid tumors, such as head and neck squamous cell carcinoma and BC [134]. The therapeutic objective of this target is to attack the tumor stroma, which is involved in oncogenesis, treatment resistance, and metastasis.

### 3.4. Immune Checkpoints Blockers (ICB)

It has been described that some BC subtypes have a dense lymphocytic infiltration, with TILs cells. A higher proportion of TILs is associated with a favorable prognosis [135,136] and, therefore, with an elevated expression of PD-L1 [137,138]. Consequently, a high proportion of TILs in TNBC and HER2+ tumors will predict a better response to PD-1 inhibitors [139], which would confirm the therapeutic potential of immune checkpoint blockade in BC patients [140]. PD-L1 is expressed not only on tumor cells but also on immune cells (activated T cells, MDSCs, B lymphocytes, monocytes, macrophages, NK cells, and DCs) and on endothelial cells [141,142,143]. The interaction between PD-1 and PD-L1 inhibits innate and adaptive immunities, which contributes to an immune evasion mechanism exploited by tumor cells [91,144]. Therefore, the disruption of this interaction has become one of the most studied therapeutic pathways in immunotherapy by the use of PD-1 inhibitors.

**Figure 2:**
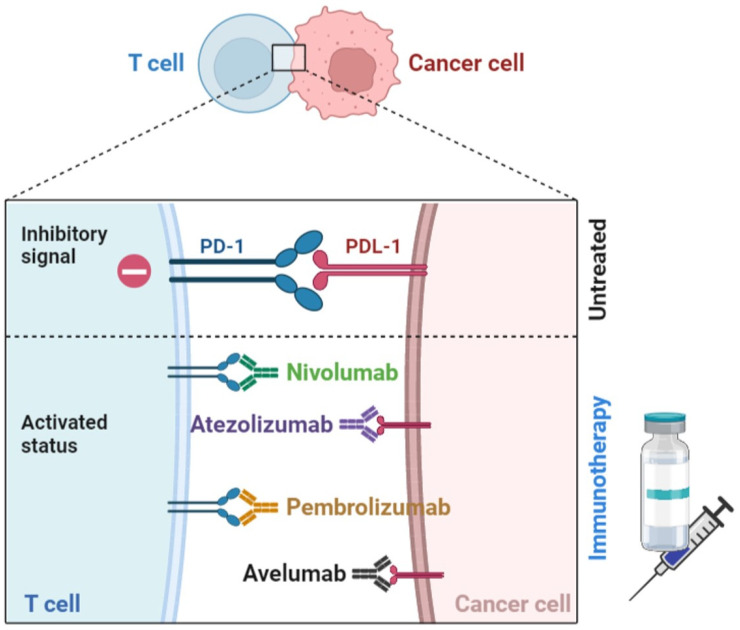
Schematic representation of anti-PD-1/PDL1: Binding of PD-1 to its ligand, PDL-1, results in suppression of proliferation and immune response of T cells. Antibody blockade of PD-1 or PD-L1 reverses this process, resulting in enhanced anti-tumor immune responses.

The blockade agents mainly studied in BC are anti-PD-1, such as nivolumab and pembrolizumab, and anti-PD-L1, including atezolizumab, durvalumab, and avelumab [144] (Figure 2). The first results of phase I a/b and phase II clinical trials have been generated, carried out in monotherapy in different BC immunophenotypes, although mostly focused on metastatic TNBC with positive PD-L1 expression, such as the KEYNOTE 12 study (NCT01848834) with pembrolizumab, the PCD4989g trial (NCT01375842) with atezolizumab, or the JAVELIN study (NCT01943461) with avelumab incorporated TNBC, HER2+ and ER+/HER2- patients, among others [97]. Currently, the only FDA-approved anti-PD-1 regimen for TNBC is pembrolizumab combined with CT, in early disease, regardless of the PD-L1 expression, based on the results of the KEYNOTE 522 trial (NCT03036488), which showed that the combination in neoadjuvant treatment and then as a pembrolizumab single agent for adjuvant treatment improved pCR and event-free survival (EFS). In the metastatic setting with PD-L1 expression, the KEYNOTE 355 trial (NCT02819518) tested the combination of pembrolizumab with CT as a first-line treatment, showing improved OS. Another FDA-approved regimen is atezolizumab with CT based on the results of the IMPASSION 130 trial (NCT02425891) for PD-L1-expressing metastatic TNBC, which demonstrated that atezolizumab with nanoparticle albumin-bound (nab)-paclitaxel prolonged PFS in patients with PD-L1-expressing metastatic TNBC compared to patients receiving placebo plus nab-paclitaxel. A variety of clinical trials are still ongoing to study alternative combinations of IBCs with other drugs in luminal tumors, HER2+ and TNBC [12,144,145,146,147,148,149,150,151].

Besides PD-L1 expression in tumors, tumor mutation burden (TMB) and deficient DNA mismatch repair genes are some biomarkers known to be associated with response to ICBs [152,153]. However, modest results have been observed in BC, where tumors are rarely hypermutated [154]. BC type 1 or 2 susceptibility genes (*BRCA1* and *BRCA2*), the most frequent hereditary germline mutated genes in BC, play critical roles in DNA repair through homologous recombination, to maintain genome integrity [154]. *BRCA1* and *BRCA2* are tumor-suppressor proteins essential for cell division, DNA replication error control, and apoptosis. They are present in approximately 5% of patients with BC and increase lifetime risk of BC 60–70% [155,156]. *BRCA 1*-associated tumors mostly show a triple negative phenotype (70–85%), with high grade, extensive lymphocyte infiltration in TME and higher mutational burden, suggesting that they generate more neoantigens to incite T-cell response [154,155]. On the other hand, luminal tumors (ER+, HER2 negative) are usually related to *BRCA 2* germline mutations. There seems to be an increased expression of PD-1 and PD-L1 within tumors that are *BRCA* mutated [154,157]. In a study by the Memorial Sloan Kettering Cancer Center, *BRCA2* alterations in tumors related to a higher TMB showed enhanced response to ICBs [152].

Platinum agents, such as cisplatin and carboplatin, and poly(adenosine diphosphate–ribose) polymerase inhibitors (PARPi), such as olaparib and niraparib, have demonstrated efficacy for the treatment of *BRCA1*-mutated BC in clinical trials in both early and advanced stages [155,157]. Monotherapy with PARPi has not shown activity outside patients with *BRCA* mutations, and they have not been studied in tumors with DNA repair defects other than *BRCA* [158]. However, the appearance of resistance to treatments invariably occurs, creating the need to explore new drug combinations to achieve more durable responses [157]. The combination of CT and anti-PD-1 demonstrated an improvement in survival in *BRCA1*-mutated tumors [157] and the combination of PARPi and ICBs has been explored in many clinical trials, showing promising results [152,156,158]. Increasing evidence shows an interaction between olaparib-induced DNA damage and the immune system: PARPi release DNA fragments, neoantigens which make tumor cells more immunogenic and more sensitive to anti-PDL-1/PD-1 immunotherapy [156,159]. 

The TOPACIO phase II clinical trial combined niraparib and pembrolizumab (NCT02657889) to treat patients with advanced or metastatic TNBC despite *BRCA* status, demonstrating safety and tolerability, with better clinical activity in *BRCA*-mutated tumors, with an ORR of 47%, a disease control rate of 80%, and a median PFS of 8,3 months in these patients [158]. The MEDIOLA phase I/II clinical trial (NCT02734004] explored the combination of durvalumab and olaparib in patients with germline *BRCA 1*- or *2*-mutated metastatic BC [156]. The rationale was based on preclinical data that suggest that PARPi might elicit an antitumor immune response. Further, 80% of patients had disease control at 12 weeks (primary endpoint), with median duration of response 9,2 months and ORR 63%. Median PFS was 8.2 months. Clinical outcomes were similar between patients with *BRCA1* versus *BRCA2* mutations and the combination was well tolerated [156]. However, both studies were carried out in a small population (47 and 30 patients, respectively) and a control group was not used. There are several ongoing studies combining other PARPi with immunotherapy agents, as found in Table 1.

Other studies are underway with CTLA-4 inhibitors, although efficacy data are more limited [136]. Ipilimumab combined with entinostat and nivolumab are currently ongoing in a phase I trial in HER2-metastatic BC. Other combinations are tremelimumab with durvalumab in HER2 negative BC patients. In a phase I trial, tremelimumab and exemestane are currently being combined in HR+ BC patients [136]. Another therapeutic target under investigation is the lymphocyte activation gene-3 receptor (LAG3), which is found expressed on activated T cells, NK cells, and DCs, and suppresses the activation and proliferation of these cells to ultimately inhibit antitumor activity. For example, eftilagimod alfa (IMP321) is an anti-LAG3 that has been evaluated in a phase II trial (NCT02614833) with paclitaxel/placebo in metastatic BC [136].

Immunotherapy has recently become established as an alternative in the treatment of cancer in general and BC in particular, combined in the vast majority of cases with other drugs to enhance their effects and avoid drug resistance. ICBs as monotherapies generate durable responses in only a sub population of patients, thus the combination regimens with other drugs should be considered. In addition, CT may have immunomodulatory effects, such as decreasing Treg levels, inducing type I IFN response by releasing tumor antigens [160] and favoring increased PD-L1 expression [161]. This combination is based on the results of the different clinical trials conducted. Additionally, many clinical trials are currently underway to evaluate the efficacy and safety of these combinations in the different subtypes of BC, as visualized in Table 2.

### 3.5. Stimulatory Molecule Agonist Antibodies

To generate an optimal immune response, the participation of co-stimulatory receptors is fundamentally required, including (but not limited to) CD27, CD28, CD40, CD134, CD137, glucocorticoid-induced TNFR-related protein, and ICOS, expressed in lymphoid cells and DCs. Their purpose is to activate the functions of these cells and suppress the immunosuppressive activity of Tregs [162]. Clinical trials are currently ongoing in different stages of the disease to study the efficacy and safety of these therapeutic targets. A phase II trial (NCT03971409) for TNBC patients currently combines an OX40 agonist, PF-04518600, with nivolumab. Similarly, the efficacy and safety of itomilumab is being determined in the phase I trial NCT03364348 in HER2+ advanced BC in combination with trastuzumab [97,145].

## 4. Myeloid-Derived Suppressor Cells as a Therapeutic Target in Breast Cancer

MDSCs are a group of cells widely studied in cancer, including BC. This is a heterogeneous population of immature, pathologically activated myeloid cells defined by their morphology, surface phenotype, and functions. MDSCs can be mainly divided into monocytic MDSCs (M-MDSCs), with a typical monocyte morphology, and granulocytic or polymorphonuclear MDSCs (G-MDSCs or PMN-MDSCs, respectively), with a morphology that resembles granulocytes [163,164,165]. In humans, M-MDSCs are CD11b+CD14+HLA-DR-/lowCD15- cells and G-MDSCs are characterized by the CD11b+CD14+CD15+ or CD11b+ CD14- CD66b+ phenotype. Cells characterized as Lin- (including CD3, CD14, CD15, CD19, CD56) HLA-DR-CD33+ are more immature groups of MDSCs, the so-called “early stage-MDSC” (e-MDSC), a term previously proposed [30,166]. In addition, the expression of both CD45 and CD33 has been considered to define MDSC subpopulations in some diseases, including cancer [167,168]. In BC settings, there are some cytokines associated with the development, differentiation, and expansion of MDSCs, including granulocyte colony-stimulating (G-CSF), macrophage colony-stimulating factor (M-CSF), GM-CSF, IL-6, IL-1β, macrophage migration inhibitory factor (MIF), and TGFβ [169,170,171]. Similarly, a set of chemokines participates to promote the recruitment of MDSCs into the TME, such as C-X-C motif chemokine ligand (CXCL)5, C-C motif chemokine ligand (CCL)1, CCL2, CCL5, or the monocyte chemotactic protein-1, among many others. Importantly, other factors that favor the recruitment of MDSCs to the main metastatic niches of BC include the S100 calcium-binding protein A8 and A9 (S100A8 and S100A9, respectively) [169,170,171].

In breast tumors, MDSCs exert their potent immunosuppressive functions through several pathways: (a) STAT3-NF-Κb-IDO, which is activated by tumor-derived IL-6 to activate STAT3 in MDSCs [171]. STAT3 modulates the expression of genes involved in inflammatory processes and promotes IDO expression, favoring the inhibition of immune surveillance, and immune tolerance by suppressing T-cell activation through the TCR and inducing the amplification of Tregs [171]. (b) STAT3/interferon regulatory factor 8 (IRF8) pathway, in which G-CSF and GM-CSF promote the low expression of IRF-8, via STAT3 and STAT5 pathways. Specifically, IRF-8 is a negative regulator of human MDSCs, whereby low levels of IRF-8 are accompanied by an increased number of MDSCs. (c) PTEN/AKT pathway, since a low PTEN expression promotes the activation of the AKT pathway (including mTOR and NF-κB pathways), and increased expression of metalloproteinases (MMPs), including MMP2, MMP13, and MMP14, to promote invasion and metastasis [169,170]. MDSCs act as progenitors of osteoclasts, promoting bone metastasis through the nitric oxide (NO) production and cross-communication with tumor cells, which represents a major problem causing high morbidity and mortality in BC patients, at least in part due to the crosstalk between MDSCs and tumor cells [171].

In recent years, a multitude of studies have been carried out, which have reported the relationship between MDSCs and BC cells. Tumor cells recruit elements, such as MDSCs, Treg, and M2 macrophages, to shape a pro-tumorigenic microenvironment that deregulates the antitumor immune responses. From a clinical perspective, the levels of circulating MDSCs have been correlated with tumor stage and metastatic disease burden in patients [170]. New research in this field establishes the relationship between elevated levels of MDSC in peripheral blood of BC patients and the prognosis of the disease, as it is associated with advanced stages, higher tumor burden, and lower PFS and OS, as well as lower response to CT, RT, immunotherapy, and targeted therapies [172]. The group of J. Markowitz demonstrates that MDSC levels in peripheral blood are associated with tumor burden of metastatic BC patients and the decreased of circulating MDSCs improved therapeutic results [173]. A study carried out by Bergenfelz et al. reported elevated circulating monocytes and M-MDSCs in patients with primary BC, loco-regional recurrence, and metastasis, who were compared with a healthy cohort of individuals [174]. Additionally, monocyte levels were altered in patients with early-stage BC, suggesting that small, localized tumors showed a systemic response affecting circulating myeloid cells during tumor development.

In addition, T-cell proliferation was suppressed in patients with early BC and those with a more advanced stage of the disease, which was positively correlated with M-MDSC levels. In the same line, the monocyte/T-lymphocyte ratio was higher in advanced BC patients than in healthy controls, suggesting that breast tumors also recruit proinflammatory cells into the TME and the ability of T cells to perform strong immune responses is low. In this study, they also highlighted that the high frequency of M-MDSCs was associated with worse prognostic disease in patients, with a high number of metastases, including lymph nodes and visceral organ [174]. In the same line, previous studies had reported that monocytes increased the invasive and metastatic potential of BC cells. Specifically, the work of our research group in the field of MDSCs in a variety of diseases, including both advanced and early-stage BCs, demonstrates that high levels of these cells in peripheral blood of patients favors an immunosuppressive microenvironment that promotes the development of metastasis and tumor progression [175]. Thus, not only M-MDSCs but also G-MDSCs in peripheral blood may be a promising, interesting biomarker to assess disease progression in BC patients, as well as a possible therapeutic target. 

Given the emerging importance of MDSCs in BC, this makes them an attractive target for further research into their functionality as a therapeutic target in this disease.

### MDSC-Targeted Immunotherapies in Breast Cancer

Currently, there are a variety of immunotherapeutic strategies in BC to target MDSCs, either reducing their number or inhibiting their immunosuppressive functions [176]. Blocking the production of cytokines and chemokines that promote the development and migration of MDSCs to the TME is one of the strategies that is being studied in BC to reduce MDSC niche [169].

Curcumin is an IL-6 inhibitor that has been previously used in a human gastric cancer xenograft model and mouse colon cancer model, demonstrating the inhibition of tumor growth, MDSCs in both blood and tumor tissue, and IL-6 levels. Furthermore, curcumin treatment polarized MDSCs to an M1 macrophage phenotype with increased expression of CCR7 and dectin 1 in vivo and in vitro [177]. Curcumin is emerging as a promising anticancer agent as it modulates mammary carcinogenesis through its effect on cell cycle and proliferation, apoptosis, senescence, and metastasis development [178]. The antitumor pathways of curcumin include the PI3K/Akt/mTOR, JAK/STAT, MAPK, NF-kb, p53, and Wnt/β-catenin [178,179]. In addition, curcumin modulates the TME, immunity, BC stem cells, and BC-related miRNAs [178].

Bone Morphogenetic Proteins (BMP4), a member of the TGFβ growth factor family, reduces the expression of G-CSF in human and mouse BCs [169]. BMP4 is a potent suppressor of metastasis development in BC. In a murine model, it has been observed that the development of breast tumors with high metastatic potential is associated with a high accumulation of MDSCs, which could be specifically induced by treatment with G-CSF/Csf3 or by G-CSF secretion from tumor sites. It is known that MDSCs are associated with poor prognosis in BC patients and therapies based on BMP4 activation and may offer a new treatment strategy in clinical settings [180]. 

r84 is an anti-VEGF inhibitor that reduces the production of IL-1β, IL-6, and CXCL1 [169]. Roland’s group conducted a study using three different BC murine models (MDA-MB-231 xenograft, 4T1 syngeneic, and a transgenic model with MMTV-PyMT mice) to study the effects of different anti-VEGF therapies, including r84, on tumor vasculature, immune cell infiltration, and cytokine levels. Specifically, r84 selectively inhibited the binding of VEGF to its receptor VEGFR2, resulting in the decrease of the levels of cytokines, such as IL-1b, IL-6, and CXCL1, the inhibition of immune suppressor cell infiltration, the increase of the DC fraction, and the downregulation of angiogenesis, which were correlated with the response to treatments. In addition, another anti-VEGF treatment, called bevazizumab, is in use for HER2-negative metastatic BC patients [181].

Sulforaphane is a MIF inhibitor [169] that blocks the pleiotropic, inflammatory, and protumor effects of that cytokine [182]. In preclinical models of BC, MIF not only promotes tumor growth and lung metastasis, but also favors the differentiation of MDSCs in TME. In vitro, pharmacological inhibition of MIF reduced the accumulation of MDSCs in the TME and blocked their differentiation. Thus, MIF inhibitors may be considered as a therapeutic target either alone or in combination with other treatments, including immunotherapy [183]. Sibylline downregulates the expression of CCR2 in MDSCs to decrease levels of this cell population. NG-monomethyl-L arginine acetate, an inducible NO synthase inhibitor, blocks the differentiation of MDSCs into osteoclasts, and may be effective in the characteristic osteolysis of MDSCs. Regarding the elimination of MDSCs, both preclinical and clinical studies are underway, most of them proposing the maturation of MDSCs [169]. Studies have been conducted to evaluate the antitumor effect of Sybilline on MDSCs. For this purpose, mice bearing mammary tumors have been used and a lower accumulation of MDSCs was observed both in blood and in the tumor tissue [184].

Activated T cells (ATCs) combined with anti-CD3 x anti-Her2/neu bispecific antibodies (aATC) have been able to eliminate MDSCs mainly via INFɣ and IL-2 [79]. Thakur et al. investigated whether ATC armed with bispecific antibodies (aATCs) could inhibit tumor growth and in turn decrease MDSC levels in a microenvironment enriched with IL-2 and INF gamma cytokines. The data obtained confirmed that aATC effectively inhibited tumor growth, also, IL-2 and INF gamma suppressed the actions and functions of MDSCs and Treg differentiation. Thus, aATCs can be targeted for treatment in BC [185].

Adoptive cellular therapy of reprogrammed tumor-sensitized immune cells includes CD25+NKT, NK, and memory T cells, and attenuates the immunosuppressive function of MDSCs due to their maturation into DCs via NKT cells through NKG2D-dependent signaling. From a clinical perspective, it has been shown that the combined injection of IL-7 and IL-15 into BC lesions after radiofrequency thermal ablation can reduce the number of MDSCs and thus inhibit tumor growth and metastasis [186].

Other strategies tested in preclinical models involve a vaccine composed of Listeria monocytogenes expressing the TAA Mage-b and c-di-GMP as a stimulatory ligand. With this, an enhanced immune response was observed due to the response of TAA-specific T lymphocytes [187]. Preclinical studies with Listeria have reported decreased levels of MDSCs on both blood and tumors, as well as enhancing T- and NK-cell immune responses, suggesting the effectiveness of Listeria immunotherapy in metastatic BC [188]. 

Clinical trials combining immunotherapy with other types of therapies in BC to target MDSC are currently underway, as shown in Table 3. Therefore, considering that MDSCs play a key role in the BC microenvironment to favor both tumor growth and metastasis, MDSC-targeting therapies may be potential treatments in clinical settings.

## 5. Conclusions and Future Perspectives

**Figure 3:**
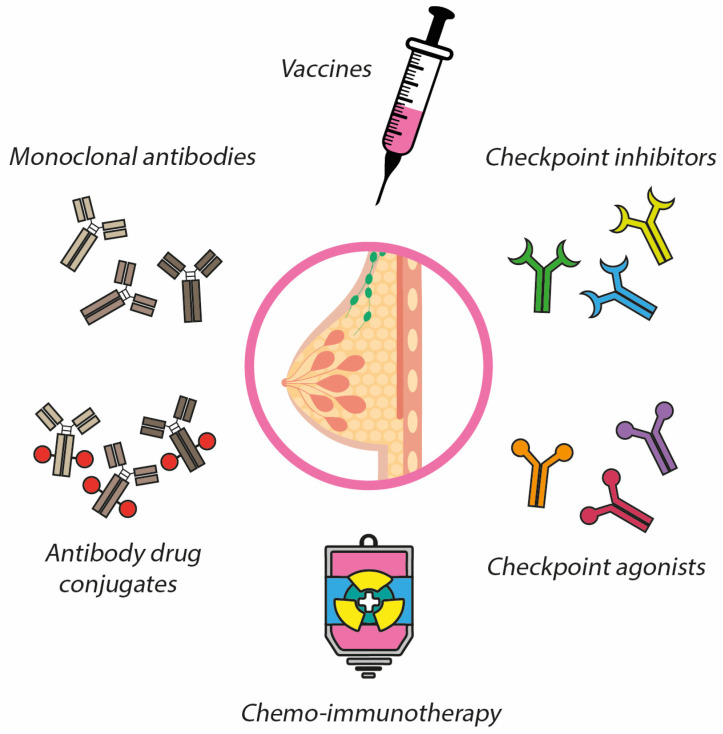
Therapeutic approaches in breast cancer settings.

Immunotherapy has emerged as a very promising therapeutic approach in cancer because it has shown to boost survival rates not only in preclinical settings, but also in oncological patients with different types of tumors. Until now, immunotherapy has not been prioritized for BC treatment because BC has been considered a poorly immunogenic tumor for many years. However, increasing evidence in recent years has suggested some immunogenic activity in different BC subtypes [189,190], exhibiting the triple-negative phenotype, the strongest immunogenicity [191], which suggests that immunotherapy may be increasingly important in the treatment of BC. In line with this notion, highly effective treatments are currently being used routinely in the clinical practice (Figure 3), including but not limited to mAbs or ADCs. However, some of those immunotherapies still need to be further investigated in terms of development and design, such as anti-cancer vaccines, which have demonstrated promising results in early clinical trials and may change the course of BC. In addition to developing new immunotherapeutic drugs, their combination with conventional BC therapies, such as hormone therapy or CT, are emerging and are considered as promising strategies to improve clinical results in terms of tumor growth, response to treatment, and survival rates.

Although immunotherapy may be an effective approach, future studies should evaluate the effects of those treatments considering that BC presents a variety of different immunological profiles. In addition, the number of cancer patients and healthy individuals recruited should be increased to validate relevant immunological biomarkers clinically [186]. This is important because the well-established biomarkers could provide a stratification of the BC patients, which are critical to the effectiveness of immunotherapy both in monotherapy and in combination. However, to determine the best strategy for these patients will be a long-term challenge. We think that the immunogenicity of BC will contribute to more effective and personalized therapeutic strategies to target the most immunogenic subtypes [170], with more favorable toxicity profiles.

Finally, it should be kept in mind that the effectiveness of immunotherapies depends on not only cancer cells, but also the immune composition of the TME, which usually is shaped by TILs, immunosuppressor cells, cytokines, CAFs, or other molecules that modulate immune responses. A better understanding of the interplay between breast tumor cells and the microenvironment will provide new directions for therapeutic strategies. Specifically, it has been described that MDSCs attenuate anti-tumor immunity to promote tumor growth and metastasis in a variety of diseases, including BC, and thereby reducing the effect of immunotherapies. For that reason, there is great interest in discovering the complexity and heterogeneity of MDSCs to develop and design new therapeutic strategies. Indeed, several therapeutic approaches have been addressed in order to modify the behavior of MDSCs, such as their downregulation or inhibition of their functions. Furthermore, in the coming years, it will be possible to assess whether the combination of MDSCs with ICIs can overcome the existing limitations of immunotherapy in cancer treatment [171]. Considering that MDSCs play an important role in the development of BC and, in turn, MDSCs can be suppressed by using different strategies, we believe that immunotherapy targeting MDSCs has a broad future perspective.

## Figures and Tables

**Table 1:** Clinical trials testing PARPi combinated with immunotherapy in *gBRCA1/2 m* breast cancer.

PARPi + Immunotherapy	Class	Settings	Clinical Trial Phase	Status	Clinical Trials Reference
Talazoparib + Avelumab	PARPi + anti-PD-L1	Metastatic solid tumor (TNBC, NSCLC, UC, CRPC)	II	Completed *	NCT03330405
Niraparib + Pembrolizumab	PARPi + anti-PD-1	TNBC or recurrent ovarian cancer	I/II	Completed Has Results	NCT02657889 (TOPACIO)
Olaparib + Atezolizumab	PARPi + anti-PDL-1	Non-HER2positive mBC	II	Active, not recruiting	NCT02849496
Olaparib + Durvalumab	PARPi + anti-PDL-1	mTBNC	II	Completed Has Results	NCT03167619 (DORA)
Olaparib + MEDI4736	PARPi + anti-PDL-1	gBRCAm HER2 negative mBC	I/II	Active, not recruiting	NCT02734004

* This clinical trial is already completed but final results have not been published yet. PARPi: Poly(ADP-ribose)polymerase(PARP)inhibitors; TNBC: Triple negative breast cancer; mBC: metastatic Breast Cancer; NSCLC: non-small cell lung cancer; UC: Urogenital cancer; CRPC: Castration-resistant prostate cancer.

**Table 2:** Ongoing clinical trials testing immunotherapy combined with chemotherapy in breast cancer.

Immunotherapy	Treatment	Additional Treatments	Settings	Clinical Trial Phase	Status	Clinical Trial Reference
Vaccines	Talimogene laherparepvec	Paclitaxel	TNBC	I/II	Active, not recruiting	NCT02779855
	Mammaglobin-A DNA	Neoadjuvant hormonal therapy	HR+ BC	IB	Recruiting	NCT02204098
	PVX-410	Alone/Combined with Durvalumab	Stage II/ III TNBC	IB	Active, not recruiting	NCT02826434
	Nelipepimut-S + GM-CSF	Trastuzumab	HER2+ BC high risk	II	Completed *	NCT02297698
Monoclonal antibodies/antibody drug conjugates	QL1209	Trastuzumab, docetaxel	Early/locally advanced ER/PR- BC	III	Not yet recruiting	NCT04629846
	Pyrotinib	Epirrubicin, cyclophosphamide͢͢͢, taxanes, trastuzumab	Moderate/high risk early BC	II/III	Not yet recruiting	NCT04290793
	Trastuzumab Deruxtecan	Pertuzumab, placebo	Metastatic	III	Recruiting	NCT04784715
	Pyrotinib	Trastuzumab, docetaxel	Metastatic	III	Recruiting	NCT03863223
	Margetuximab	Capecitabine, vinorelbine, Gemcitabine, Eribulin	Metastatic	III	Active, not recruiting	NCT02492711
		Paclitaxel, pertuzumab	Neoadjuvant	II	Recruiting	NCT04425018
Anti-PD-1	Nivolumab	Nanoparticle albumin-bound paclitaxel ± Gemcitabine or Carboplatin	Metastatic	II	Recruiting	NCT02309177
	Nivolumab	Paclitaxel, cyclophosphamide, endrocine therapy, anthracycline	Metastatic	III	Active, not recruiting	NCT0419066
	Nivolumab	Low dose doxorubicin, cisplatin	Metastatic	II	Recruiting	NCT04159818
	Pembrolizumab	Carboplatin	Metastatic	II	Recruiting	NCT03213041
	Pembrolizumab	Abemaciclib	Metastatic	Ib	Active, not recruiting	NCT02779751
	Pembrolizumab	Trastuzumab emtansine	Metastatic	Ib	Active, not recruiting	NCT03032107
Anti-PD-L1	Atezolizumab	Carboplatin	Metastatic	II	Active, not recruiting	NCT03206203
	Durvalumab	Hypofractionated RT, Tremelimumab	Metastatic	I	Active, not recruiting	NCT02639026
	Avelumab	Palbociclib and Fulvestrant	Metastatic	II	Active, not recruiting	NCT03147287

* This clinical trial is already completed but final results have not been published yet. BC: Breast cancer; ER/PR-: Estrogen and progesterone receptor-negative; GM-CSF: Granulocyte-macrophage-colony stimulating factor; HER2+: Human epidermal growth factor receptor 2positive; HR+: Hormone receptor-positive; PD-1: Programmed cell death 1; PD-L1: Programmed cell death ligand 1; PVX: Peptide vaccine; RT: radiotherapy; TNBC: Triple negative breast cancer.

**Table 3:** Myeloid-derived suppressor cell-targeting therapies in breast cancer clinical trials.

Therapy	Combination Therapy	MDSC Target	Additional Tumors Tested	Clinical Trial Phase	Status	Clinical Trial Reference
Entinostat	Nivolumab	Class I HDCA	-	I	Active, not recruiting	NCT02453620
IPI-549	Nivolumab	PI3K	NSCLC, SCCHN, AdC, MEL, MES	I/Ib	Active, not recruiting	NCT02637531
IPI-549	Tecentriq + Abraxane	PI3K	-	II	Active, not recruiting	NCT03961698
Reparixin	Paclitaxel	CXCR2	-	II	Completed	NCT02370238
AB928	IPI-549, PLD, NP	AzaR and AzbR	Ovarian	I/Ib	Completed	NCT03719326
PD-0360324	Avelumab	CSF-1	A variety of advanced tumors (including BC)	Ib/II	Active, not recruiting	NCT02554812
Ciclophosphamide/Decitabine/carboplatn/paclitaxel/Doxorubicin	Pembrolizumab	PD-1	-	II	Active, not recruiting	NCT02957968
Leronlimab	Carboplatin	CCR5	-	Ib/II	Active, not recruiting	NCT03838367
Imiquimod	Paclitaxel	TLR7	-	II	Completed	NCT00821964

AdC: Adrenocortical carcinoma; BC: Breast cancer; CCR5: C-C motif chemokine receptor 5; CSF-1: colony-stimulating factor 1; CXCR2: C-X-C motif chemokine receptor 2; HDCA: Histone deactylase; IPI-549: Inhibitor of PI3K-γ-549 (Eganelisib); MDSC: myeloid-derived suppressor cell; MEL: melanoma; MES: Mesothelioma; NP: Nanoparticle albumin-bound paclitaxel; NSCLC: non-small cell lung cancer; PD-1: Programmed cell death 1; PDL: pegylated liposomal doxorubicin; PI3K: phosphatidylinositol 3-kinase; SCCHN: Squamous cell cancer of head and neck; TLR7: Toll-like receptor 7.
